# Serogrouping and antibiotic resistance of *Escherichia coli* isolated from broiler chicken with colibacillosis in center of Algeria

**DOI:** 10.14202/vetworld.2017.830-835

**Published:** 2017-07-28

**Authors:** Zehor Halfaoui, Nabil Mohamed Menoueri, Lyes Mohamed Bendali

**Affiliations:** 1 Veterinary Institute, University of Blida 1, Road of Soumaa, BP 270, Blida 09000, Algeria; 2 Medical Analysis Laboratory, Miliana, BP 44200, Algeria

**Keywords:** Algeria, antibiotic resistance, broilers, colibacillosis, *Escherichia coli*, serogrouping

## Abstract

**Aim:**

Colibacillosis is considered as one of the major bacterial infections in avian pathology. The excessive use of antibiotics reduced their effectiveness, which eventually led to the risk of emergence of antibiotic resistance. The aim of this study was to isolate, identify and serotype the pathogenic *Escherichia coli* strains and to determine their antibiotic susceptibility.

**Materials and Methods:**

A total of 180 samples from different organs of broilers with colibacillosis lesions were collected (liver, spleen, lung, and heart) in center of Algeria. The isolation and identification of *E. coli* were carried out using conventional techniques. Then, these strains were serotyped and tested over 13 antibiotics.

**Results:**

A total of 156 strains of *E. coli* were isolated. Serotyping results showed that 50 strains belong to 3 serotypes (23 for O1, 11 for O2, 16 for O78) which represent 32% of isolates. The antimicrobial susceptibility test, presented high level of resistance to tetracyclines (94.12%), flumequine (91.5%), sulfamethoxazole-trimethoprim (88.89%), enrofloxacin (86.27%), nalidixic acid (85.62%), ampicillin (83.01%) and doxycycline (75.81%), medium level resistance to chloramphenicol (39.22%), and amoxicillin-clavulanic acid (43.13%). All the strains were susceptible to cefotaxime, excepting three, which presented an extended spectrum β-lactamase (ESBL). In addition, the results of multi-resistance showed that all the strains were resistant at the minimum to two antibiotics and 66.66% of strains were resistant to at least seven antibiotics.

**Conclusion:**

The antibiotic resistance continues to rise at an alarming rate, and the emergence of ESBL is considered as a threat for public health.

## Introduction

*Escherichia coli* is widely used as an indicator for selection pressure imposed by antibiotic use and for resistance problems to be expected in pathogens. These bacteria are mainly responsible for causing colibacillosis, which is considered as a major health concern in poultry. This disease contributes significantly to economic losses in the poultry industry in Algeria, as it leads to mortality, seizures and reduction of performance in slaughterhouses [1,2]. Unlike in mammals cases, *E. coli* in poultry is relatively implicated in digestive pathology but contributes in various syndromes evolving under septicemic or localized form, chronic respiratory disease, omphalitis, synovitis, coligranulomatosis, salpingitis, grouped under the name avian pathogenic *E. coli* (APEC) [3]. The diagnosis of avian colibacillosis is primarily based on the clinical signs and characteristic lesions at necropsy, such as airsacculitis, perihepatitis, and pericarditis. If colibacillosis is suspected, isolation and identification of the pathogen will be recommended [4].

The isolation of *E. coli* strains from a characteristic lesion is considered as challenging during its identification as pathogenic or nonpathogenic. *E. coli* is a regular host of the digestive tract of poultry, the isolation of a nonpathogenic strain cannot be totally excluded, and the mechanism by which APEC cause infection are largely unknown [5]. It is, therefore, necessary to improve the characterization of potentially pathogenic strains by serotyping [4].

More than 1000 serotypes are known, but only a few are considered as important in avian pathology, earlier studies by Sojka and Carnaghan identified the serotypes O1, O2, O35, and O78 as the most dominated [6]. However, recent studies have shown that the serotypes O1, O2, and O78, are widely spread and represent 15-61% of the isolates, yet other types still exist [7].

Avian *E. coli* is considered as secondary pathogens; however, recently they are considered, in Algeria, as one of the most important causes of economic losses in the poultry sector. This study was undertook due to the hygienic and economic impacts of colibacillosis and the risk of emergence of resistance to antibiotics.

## Materials and Methods

### Ethical approval

No chickens were harmed during collection of samples.

### Collection of samples

The study was conducted at the wilaya de Ain Defla (center of Algeria: Latitude 36.25, longitude 2.09). The sites were chosen based on their veterinary practices with activities mainly focused on poultry farming. Three veterinarians participated in this study, and they received clinical cases and performed poultry autopsies.

The autopsy was performed according to the standard procedure of poultry autopsy. The organs with characteristic lesions were removed and they included liver, heart, lung, and spleen. The removed organs were placed in sterile bottles, immediately transported in ice-packed containers to the bacteriological laboratory and kept at −20°C. The samples were collected between January and September 2014 and analyzed at the microbiology laboratory.

### Isolation and identification of E. coli

The organs were flamed using a Bunsen burner and cut into small pieces. Each piece was placed in a tube of pre-enrichment broth brain heart infusion broth (heart-brain broth) (Pasteur Institute of Algeria, IPA). After 24 h of incubation at 35°C, a drop from the broth was collected and inoculated on Hecktoen agar (IPA). The various inoculated plates were incubated at 35°C for 18 h. The appeared colonies were observed macroscopically; salmon yellow colonies were selected and seeded on nutrient agar (IPA) and incubated at 35°C for 18 h. The pure colonies were characterized microscopically by motility observation and by Gram-staining. Then, each colony was subsequently identified with an API 20 E system kit (*Enterobacteriaceae*) (Bio-Merieux, France).

### Serogrouping

After the isolation and identification procedures, *E. coli* strains were serogrouped by rapid slide agglutination tests using sera antibodies that directly correspond to the O antigens. The agglutination reaction occurs when the specific antibodies present in the serum bind with bacterial antigens and it can be macroscopically observed. In this study, only O1, O2, and O78 antigens were tested as they are considered as the most common pathogenic *E. coli* strains. The reagents used are based on specific antibodies obtained from immune sera of rabbits (Bio vac, Animal Health, France).

### Antimicrobial susceptibility test

Antibiotic sensitivity was determined by disc diffusion method on Mueller-Hinton agar medium (IPA), according to the guidelines of National Committee for Clinical Laboratory Standards recommended by WHO. *E. coli* ATCC 25922 strain was used for quality control. The following 13 antibiotics commonly used in poultry farming were applied: Ampicillin (10 ug), amoxicillin + clavulanic (AMC) acid (20/10 ug), colistin sulfate (10 ug), tetracycline (30 μg), nalidixic acid (30 μg), enrofloxacin (5 µg), flumequine (30 µg), Gentamycin (10 ug), chloramphenicol (30 μg), sulfamethoxazole + trimethoprim (1.25/23.75 µg), doxycycline (30 IU), nitrofurantoin (300 µg), and cefotaxime (CTX) (30 µg). The diameters of inhibition zones were interpreted by referring to the table of *Enterobacteriaceae* as recommended by the Standardization of Susceptibility to the National Scale Human and Veterinary (2011).

### Search of β-lactamases extended spectrum (ESBL)

ESBLs can hydrolyze penicillins, cephalosporins 1^st^, 2^nd^, 3^rd^, and 4^th^ generation and monobactams and are generally susceptible to carbapenems. The ESBL A class is inhibited by inhibitors of β-lactamases, as clavulanic acid.

The search for the ESBL was performed in standard conditions of susceptibility testing by filing AMC disk (AMC 20/10 ug) to 30 mm (center to center) of a cephalosporin disk 3^rd^ generation, CTX (30 µg) on Muller-Hinton agar (IPA), the plates were incubated for 18 h at 35°C. The detection of ESBL is conventionally based on the observation of so-called synergy “in champagne cork” between the AMC and CTX discs.

## Results

In total, 180 samples were taken from different organs (heart, liver, lung, and spleen) with characteristic lesions in which colibacillosis was inspected. The presence of *E. coli* was confirmed in 156 samples, representing 86.66% of pathological specimens. After the identification of *E. coli*, the strains were serotyped with serums O1, O2, and O78 and their antibiotic susceptibility was tested. While 153 out of 156 from the isolated strains are reported in Table-1, the remaining strains are studied separately as they had presented an ESBL profile.

**Table-1:** Antibiotic resistance and serogroups of 153 *Escherichia coli* strains isolated.

Antibiotics	Number of resistance strains (%) n=153				

	O78	O1	O2	Others	Total
Tetracycline	15 (93.7)	21 (91.3)	11 (100)	97 (94.1)	144 (94.1)
Flumequine	15 (93.7)	21 (91.3)	10 (90.9)	94 (91.2)	140 (91.5)
Sulfamide+trimethoprim	10 (62.5)	20 (86.9)	11 (100)	95 (92.2)	136 (88.8)
Enrofloxacin	12 (75)	19 (82.6)	10 (90.9)	91 (88.3)	132 (86.2)
Nalidixic acid	16 (100)	20 (86.9)	10 (90.9)	85 (82.5)	131 (85.6)
Ampicillin	12 (75)	18 (78.2)	11 (100)	86 (83.5)	127 (83)
Doxycycline	12 (75)	17 (73.9)	9 (81.8)	78 (75.7)	116 (75.8)
Amoxicillin+clavulanic acid	5 (31.2)	10 (43.4)	7 (63.6)	44 (42.7)	66 (43.1)
Chloramphenicol	9 (56.2)	9 (39.1)	4 (36.3)	38 (36.8)	60 (39.2)
Colistin sulfate	0 (0)	1 (4.3)	0 (0)	9 (8.7)	10 (6.5)
Gentamycine	1 (6.2)	0 (0)	1 (9)	1 (0.9)	3 (1.9)
Nitrofurantoide	0 (0)	0 (0)	0 (0)	0 (0)	0 (0)
CTX	0 (0)	0 (0)	0 (0)	0 (0)	0 (0)
Total number of isolates	16	23	11	103	153 (100)

CTX=Cefotaxime

The results of antibiotic resistance of 153 *E. coli* strains are presented in Table-1. Oxytetracycline is the one who present the highest level of resistance with a rate of 94.12% followed by flumequine (91.5%), sulfamethoxazole + trimethoprim (88.89%), enrofloxacin (86.27%), ampicillin (83.01%), nalidixic acid (81.04%), and doxycycline (75.16%). Chloramphenicol (39.22%) and AMC acid (43.1%) present medium level of resistance. Low resistance rates were observed to colistin sulfate (6.54%) and gentamicin (1.96%). All strains (100%) are sensitive to nitrofurantoid and CTX.

Multidrug resistance is considered as a real threat, as 100% strains of 153 of *E. coli* isolates were resistant to at least two antibiotics and 98.7% of strains were resistant to at least three antibiotics. Most strains (92.8%) were resistant to five antibiotics, and about half (43.1%) were resistant to eight antibiotics and 16.3% were resistant to nine and 10 antibiotics. The results are shown in Table-2.

**Table-2:** Strains of *Escherichia coli* showing multidrug resistance.

Number of antibiotics	Number of strains n=153	Rates of resistance strains
2	2	1.3
3	5	3.26
4	4	2.61
5	16	10.45
6	24	15.68
7	36	23.52
8	41	26.79
9	24	15.68
10	1	0.65

A total of 48 resistance profiles were obtained in our study, and the 10 common ones are reported in Table-3.

**Table-3:** The most frequent antibiotic resistance patterns of *Escherichia coli* isolates.

Resistance patterns	Designation	Number of strains (%)
Amp, Amc, OT, Ac.N, SXT, Cs, Ch, DO, ENR, UB	A	1 (0.75)
Amp, Amc, OT, Ac.N, SXT, Ch, DO, ENR, UB	B	19 (12.41)
Amp, Amc, OT, Ac.N, SXT, DO, ENR, UB	C	18 (11.76)
Amp, OT, Ac.N, SXT, Ch, DO, ENR, UB	D	17 (11.11)
Amp, OT, Ac.N, SXT, DO, ENR, UB	E	17 (11.11)
Amp, Amc, OT, Ac.N, SXT, ENR, UB	F	7 (4.57)
Amp, OT, Ac.N, SXT, ENR, UB	G	5 (3.26)
OT, Ac.N, SXT, DO, ENR, UB	H	8 (5.22)
OT, Ac.N, DO, ENR, UB	I	5 (3.26)
OT, Ac.N, SXT, ENR, UB	J	4 (2.61)

Amp=Ampicillin, Amc=Amoxicillin/clavulanicacid, OT=Tetracycline, Ac.N=Nalidixic acid, SXT=Trimethoprim-sulfamethoxazole, DO=Doxycycline, ENR=Enrofloxacin, UB=Flumequine, CS=Colistin sulfate, Ch=Chloramphenicol

Out of 156 isolated *E. coli*, only three strains (154, 155, and 156) showed a ESBLs. These *E. coli* ESBL strains had resistance to several antibiotics families inter alia, the β-lactams, tetracyclines and quinolones (Table-4).

**Table-4:** Resistance and sensitivity of the three strains with ESBL.

Antibiotic	E 154	E 155	E 156
CTX	S	S	S
AMC acid	S	S	S
Ampicillin	R	R	R
Gentamycin	S	S	S
Nalidixic acid	R	R	R
Trimethoprim-sulfamethoxazole	S	R	S
Colistin sulfate	S	S	S
Chloramphenicol	S	S	S
Doxycycline	R	R	R
Enrofloxacin	R	R	R
flumequine	R	R	R
Nitrofurantoin	S	S	S
Tetracycline	R	R	R

S=Sensitive, R=Resistant, CTX=Cefotaxime, AMC=Amoxicillin+clavulanic, ESBL=Extended spectrum β-lactamase

## Discussion

A total of 180 samples were taken from organs in which colibacillosis was inspected; the presence of *E. coli* was confirmed in 156 samples with a percentage of 86.66%. The other isolates involved other germs; some of them were identified as *Klebsiella*, *Enterobacter*, and *Salmonella*.

Serogroups O1, O2, and O78 represented 32% of the isolates. The first studies by Sojka and Carnaghan [6] on avian *E. coli* indicated that the most common serogroups were O1, O2, O35, and O78. Meanwhile, the latest studies confirmed that the serotypes O1, O2, and O78 are highly pathogenic and widely spread as they represent from 15% to 61% of the isolates, although others are also present [7].

In this study, the serogroup O1 represented 15% of the isolates. These results were similar to those of Messai *et al*. [8] and Aggad *et al*. [9] and as their percentage of this serogroup were of 15% and 14%, respectively. The O2 serogroup represented 7% and serogroup O78 represented 10% of the isolates, yet 68% of the isolated strains belonged to other seogroups. The serogroup is not a predictable value, as some of *E. coli* strains can be untypable pathogens [2]. In addition, Blanco *et al*. [10] indicated that many strains were not serogrouped as O1, O2, and O78; however, they were a part of other precarious serogroups in avian pathology (O8, O15, O18, O35, O109, O115, and O116).

In our results, the greatest resistance was observed to tetracycline with a rate of 94.12%. Similar results were presented by Aggad *et al*. [9] and Benameur *et al*. [11] in the western Algerian with resistance rates of 87% and 90.4%, respectively.

Other studies, such as those by Cheikh [12] and Rahimi [13], have reported *E. coli* strains with distinctively high resistance rates to tetracycline of 98.15% and 85.1%, respectively. This could be due to the excessive use of this molecule, either as prophylactic, curative, or as a growth factor. Bacterial resistance to tetracycline is of plasmid nature and the existence of a wide variety of genetic determinants leads to persistent acquisition of resistance genes by conjugation or transformation [14].

The existence of tetracycline medicated feed as growth factors in the Algerian market was banned. For several years, the positive effects of this practice were highlighted, while the side effects were undetectable, yet some studies focused on them. In 1975, a study done by Levy *et al*. [15] assessed that the effect of the introducing of low doses of tetracycline in poultry feed on the intestinal flora showed that the number of resistant bacteria was higher in those who only received food supplemented with tetracycline. Yet, the upsurge of resistant bacteria in the intestinal flora was not only linked to the animals but also the farm’s environment.

Resistance rate to doxycycline was high in this study (75.16%). Although doxycycline was recently introduced in the Algerian market, yet its resistance rates overlapped with those of prior antibiotics. Moreover, Bartlett *et al*. [16] indicated that the proteins used to code resistance to tetracycline are used with doxycycline which is referred to as cross-resistance.

The resistance rates to ampicillin in our study (83.01%) correspond to the results obtained by Messai *et al*. (89%) [8], and those obtained by Cheikh in Senegal (74.08) [12].

The rate of resistance to trimethoprim sulfa’s in our study was 88.89%, is correlated with the results of Messai *et al*. [8] 82%, Aggad *et al*. [9] 70%, and Benameur *et al*. [11] 70.2%. This association is widely used in avian pathology, particularly in the nonspecific treatment of coccidiosis, and to colibacillosis, which could explain the high rates of resistance.

The quinolone resistance was high in this study (85.62% for nalidixic acid and 91.5% for flumequine). Similarly; Rahimi [13] reported a higher resistance rate to these molecules (97.7% for nalidixic acid and 81.8% for flumequine). While the resistance rate to enrofloxacin was 86.27%, Benameur *et al*. [11], and Rahimi [13] had found resistance to enrofloxacin of 69.3% and 79.2%, respectively.

The high rates of resistance to the quinolone antibiotics can be explained by the excessive use of these antibiotics as they are widely spread at affordable prices, which eventually are considered as abusive and inadequate.

Fluoroquinolones are considered as one of the new classes of antimicrobials with strong activity against Gram-negative bacteria. Unfortunately, the resistance rates to these molecules have highly increased as they are misused in poultry, which may ultimately lead to cross-resistance to the fluoroquinolones used in human cases [17], as they are only used as an alternative in the treatment of certain infectious diseases in humans. Likewise, it is highly recommended in Europe that these antibiotics should be reserved as second-line in curative treatments [18].

Chloramphenicol resistance rates were relatively high 39.22%. This may be due to the persistence of previous resistances or exposes illegal usage of this molecule as it is prohibited in breeding.

Resistance to colistin 6.54% was low. The resistance of Gram-negative bacteria to colistin is considered as uncommon and exceptional; it is chromosome type, so the mutation is rare. Furthermore, studies by Maure *et al*. showed that this resistance is phenotypic or adaptive and reversible: It corresponds to an alteration of the architecture of the bacterial wall. Furthermore, it is to slow onset [19].

Gentamicin has the lowest resistance rates (1.96%) because this molecule is not used in veterinary medicine. All 153 *E. coli* strains were sensitive to CTX and nitrofurantoin, as these molecules are not used in avian pathology.

Multidrug resistance appears as a veritable problem, in our observations, 98.7% of the strains are resistant to at least three antibiotics, 92.81% are resistant to five antibiotics, 82.35% are resistant to six antibiotics, 43.13% are resistant to eight antibiotics, and 16.33% are resistant to nine and 10 antibiotics.

The high rate of multidrug resistance indicates the abusive use of antibiotics in Algeria. Many antibiotics are administered concomitantly through prophylaxis or treatment, increasing the risk of multiple resistances. Failure to achieve the orientations susceptibility test involves the multiplication of these practices and the development of resistance genes. It also leads to coresistance phenomenon, leading the development of genuine clones resistant to many antibiotics.

The resistance patterns demonstrated the evidence of this phenomenon, the most commonly used antibiotics in poultry had the largest resistance patterns in our study, this might be responsible for the therapeutic failures in colibacillosis, and this resistance problem led to major health concerns. Concern about antibiotic resistance and its transmission to human pathogens is important [20].

A study done by Dierikx *et al*. [21] supposed that the excessive use of antibiotics in chicken meat could explain (by selection or coselection) the high prevalence of broiler with acquired ESBL. Moreover, a study done by Persoons *et al*. [22] determined that the use of amoxicillin and enrofloxacin is considered as a risk factor statistically significant for the high level of resistance to ceftiofur in chicken meat.

Moreover, the prevalence of ESBL in chicken meat was also raised on farms using no antibiotics during the production period, which indicates that other factors could influence the occurrence of ESBL, like infection control measurements and the usage of antibiotics in breeding [21].

In our study, the 3 positive ESBL strains had shown resistance to tetracycline, doxycycline, as well as ampicillin and quinolones. Since bacteria are capable of producing, these hydrolyzing enzymes of penicillins and cephalosporins are often resistant to most of the antibiotics families [23].

A study done by Meguenni *et al*. [24] isolated, different strains from avian colibacillosis lesions from different farms in the center of Algeria showed that 11 strains from the isolates belonged to ESBL blaCTX-M1 group.

In the Netherlands, a study by Leverstein-Van Hall *et al*. [25] revealed that 35% of ESBLs produced by *E. coli* were responsible for human infections. In addition, it was also indicated that chicken meat was contaminated by 94% of *E. coli* ESBL producers resistant *Enterobacteriaceae*, as 19% of their plasmids carried the encoding genes of these enzymes.

Therefore, the transfer of bacterial resistance genes of an animal origin to those of a human origin, and vice versa could be biologically plausible. At present, the evaluation and quantification of these genes flow between human and animal bacterial populations are not conducted [26].

## Conclusion

Multiresistance *E. coli* strains and the emergence of ESBL are a real global threat. Thus, increasing awareness about antibiotic resistance and promoting rational use of antibiotics are the key to combating infectious diseases related to human’s health and animal’s health, as it is not sufficient to quantitatively reduce antibiotic consumption but to qualitatively improve their usage.

## Authors’ Contributions

NH performed the field work, collect the samples and analyze them. LMB has supervised the laboratory work. NMM read and approved the final manuscript. All authors read and approved the final manuscript.
